# Supplementary material for: Chromothripsis during telomere crisis is independent of NHEJ, and consistent with a replicative origin
Source: Genome Res. 2019 May;29(5):737–49. doi: 10.1101/gr.240705.118 (PMC6499312; doi:10.1101/gr.240705.118)
Supplement: Supplemental Material [file supp_gr.240705.118_Supplemental_file_1.zip › contigs/annotated_contigs/DB56/contig.2.DB56_length_382_mean_cov_9.35602094241.docx]

**DB56_length_382_mean_cov_9.35602094241**

```
TGAACAGACGATACACATTTAAAAAAATAACAACACAAGGATCAACCAGACTTGGGAAAAAATCGAAA|ACC|TTACAGTTGAATGACA
 >chrUn_gl000220:138722-138793 + E=3e-31                             >chr14:107187331-1
AAGCCACATGATCTAATTTTAAAATAGACAAAGACCTGTATAGAAAGAAGCCTCATCTAAAAATAAATATAAAAAGTGATCGGCATGAT
07187645 - E=1e-159
TTTTAGGTATATGTATGTTTAATATTCAAAAAATACTATATCCTGTTAGAATGGGTAAAATACATATTTTAGACAGTACCAAATGGTGA

TGAGGAAGTTGAAAAACAGGAACGTTCACTCCTTGCGGGTGGGAAGAGGATGCTGAAGAGGCACATACACAACTACAGTTAGTACTTTA

TTGTATATTTCAGAATAGCTAGAAGATA
```
